# Editorial: Drivers of host-microbiome interactions in the rhizosphere

**DOI:** 10.3389/fpls.2023.1294388

**Published:** 2023-09-27

**Authors:** Olivera Topalović, Kadri Koorem, Rutger A. Wilschut

**Affiliations:** ^1^ Department of Biology, Section of Terrestrial Ecology, University of Copenhagen, Copenhagen, Denmark; ^2^ Laboratory of Nematology, Wageningen University, Wageningen, Netherlands; ^3^ Department of Botany, Institute of Ecology and Earth Sciences, University of Tartu, Tartu, Estonia

**Keywords:** rhizobiome, global change drivers, host-microbe associations, multitrophic interactions, rhizosphere, sustainable agriculture, plant health and crop protection

Plant performance is tightly linked with the composition and activity of organisms inhabiting the rhizosphere, jointly forming the *rhizobiome*. The rhizobiome typically consists of microbiota (bacteria, fungi and protists) and small soil animals (nematodes and microarthropods), which all directly or indirectly interact with plants and affect plant performance (Berendsen et al., 2012; Philippot et al., 2013; Topalovic and Geisen, 2023). However, studies on individual rhizobiome members only allow to underpin the specific mechanisms by which they interact with the plant. In nature, rhizobiome members form complex interactions among themselves and with plants, which are also influenced by external factors such as soil nutrients or extreme weather events (see e.g., Hu et al., 2023). Understanding how interactions between plants and rhizobiome members are altered by changing biotic or abiotic conditions has become an important topic for plant and soil scientists. With this Research Topic, we firstly aimed to deepen the understanding of drivers of plant-rhizobiome interactions and draw the attention to the diversity of rhizobiome members that are currently studied. Secondly, we aimed to find links between the studies in order to support our argument that, although nature is very complex, it is also very much interconnected. As plant scientists *sensu lato*, our job is to study this interconnectedness on local and global scales in order to better understand plant health in natural and agricultural ecosystems.

Studies examining plant-rhizobiome interactions mostly focus on biotic communities occurring either inside or outside the roots. In their study, Lan et al. explored microbial relationships between these environments by analyzing bacterial and fungal communities inhabiting four compartments in or surrounding rubber trees: bulk soil, rhizosphere soil, the root surface (rhizoplane), and the endosphere. Their results demonstrate that, as expected, rhizosphere communities are a subset of bulk soil communities, but rhizoplane communities consist of both rhizosphere and endosphere microbes. They further demonstrate that endosphere bacterial communities are most influenced by environmental variables such as phosphorous (P), potassium and nitrogen, while endosphere fungal communities are mostly driven by plant genetic variables.

In addition to understanding how the rhizobiome is shaped by the plant, it is important to examine the impacts of key rhizobiome members on plant physiology, as changes in plant traits may in turn reshape rhizobiome community composition. Huang et al. studied how two species of ectomycorrhizal truffles (*Tuber*) affected the growth and physiology of *Castanopsis rockii* seedlings and bacterial communities in the rhizosphere. Their study shows that colonization by both fungi improved key leaf traits (e.g., leaf photosynthetic rate) and increased bacterial richness in the rhizosphere. Yet, the authors also observed species-specific effects of truffle species on plant physiology, in line with their distinct effects on bacterial communities, indicating that ectomycorrhizal fungi play an important role in shaping rhizosphere communities.

Similarly to Huang et al., Han et al. demonstrated species-specific effects on plant-microbe interactions, when studying arbuscular mycorrhizal (AM) fungi in the roots of poplar (*Populus*) species. They demonstrated that in parallel with changes in soil nutrient levels, AM fungal communities in roots changed within a year, being more species-rich and diverse in fall than in spring. They also reported that AM fungal communities vary between poplar species and between life stages within species. The authors suggest that this variation can be related to the variation in root structures, but this needs further research. Additionally, it needs to be considered, that as poplar species are simultaneously colonized by arbuscular mycorrhizal fungi, understanding the dynamics of entire fungal communities can be key for unraveling plant-microbe interactions with these host plants.

Knowledge on which rhizobiome members promote plant performance may be used to modify rhizobiome communities and enhance plant health. Aiming for more sustainable avocado (*Persea americana*) production, Villar-Moreno et al. studied the potential of synthetic community assembly of bacterial *Pseudomonas chlororaphis* strains to protect avocado roots against the phytopathogenic fungus *Rosellinia necatrix*. Combining *in vitro* and *in vivo* experiments, they showed that a synthetic community of three *P. chlororaphis* strains, extracted from avocado rhizospheres, successfully colonized avocado roots and suppressed *R. necatrix* infection. However, pathogen suppression by the synthetic community was as strong as that of an already-known pathogen-suppressive *P. chlororaphis* strain, leaving the question whether synthetic communities can provide additional pathogen suppression effects, open for now.

The importance of understanding how we can use microorganisms to promote plant performance under abiotic stressors is more important than ever. To advance this knowledge, Ji et al. looked at the transcriptional changes in roots and leaves of wheat plants treated with beneficial rhizobacteria *Bacillus velezensis* JC-K3 and *Bacillus subtilis* HG-15 in ambient conditions and under salt stress. The authors reported that the genes that were differentially expressed in treatments with bacteria are associated with the metabolism of carbohydrates, amino acids, secondary compounds and signal transduction pathways. They suggest that the rhizobacteria used in their study may have ameliorative effect on wheat in case of salt stress, by promoting growth and improving disease resistance, as seen by the expression of specific genes important for these functions.

In areas where soil moisture is limited, plant access to nutrients, especially P, is particularly challenging. In a review article, Abbasi discussed different adaptations that plants have evolved to scavenge for P under drought. Abbasi also discussed different abiotic and biotic factors that improve or reduce P uptake by the plant, pointing out that soil fungal communities play an essential role in P availability to the plant, sometimes leading to increased plant pathogen resistance. Moreover, the author summarized the evidence for contributions of P-solubilizing bacteria to plant P acquisition and the effects of other rhizobiome members on the establishment of these bacteria. Abbasi proposed that future studies should focus on exploring the putative role of P-solubilizing bacteria in plant adaptation to arid environments, which become more widespread under climate change.

As seen from the variety of studies in this Research Topic, plant performance is indisputably affected by many factors that shape the rhizobiome. In accordance with the nature and specificity of plant-rhizobiome interactions, studies in natural ecosystems such as forests are more focused on exploring the importance and drivers of microbial interactions with the plants, while studies in agricultural settings are focused on exploring the application of microbial communities to enhance plant performance. We believe that each of these study types is important to enhance our understanding on different aspects of plant-rhizobiome interactions and encourage research to combine the knowledge obtained from natural and agricultural systems to advance current understanding in host-microbe interactions in the rhizosphere.

## Author contributions

OT: Writing – original draft, Writing – review & editing. KK: Writing – original draft, Writing – review & editing. RW: Writing – original draft, Writing – review & editing.
